# Apulo-Calabrese and Crossbreed Pigs Show Different Physiological Response and Meat Quality Traits after Short Distance Transport

**DOI:** 10.3390/ani8100177

**Published:** 2018-10-10

**Authors:** Gizella Aboagye, Stefania Dall’Olio, Francesco Tassone, Martina Zappaterra, Salvatore Carpino, Leonardo Nanni Costa

**Affiliations:** 1 Department of Agricultural and Food Sciences (DISTAL), Division of Animal Sciences, University of Bologna, Viale Fanin 46, 40127 Bologna, Italy; stefania.dallolio@unibo.it (S.D.); francesco.tassone2@unibo.it (F.T.); martina.zappaterra2@unibo.it (M.Z.); leonardo.nannicosta@unibo.it (L.N.C.); 2 Associazione Regionale Allevatori of Calabria Region, 88046 Catanzaro, Italy; carpinos@libero.it

**Keywords:** pigs, local breed, Apulo-Calabrese, transport, short distance, blood parameters, meat quality

## Abstract

**Simple Summary:**

Transport is a stressful procedure that can affect adversely the welfare of pigs and pork quality. It is well known that response to the journey is influenced by the genetic type. However, very little is known on the response of local breeds to the transport procedures despite the increasing interest in the welfare of animals during transport. The objective of this study was to investigate the effect of short distance transport on behaviour, blood parameters and meat quality traits of Apulo-Calabrese (local Italian pig breed) and crossbreed [Duroc × (Landrace × Large White)] pigs. Statistical analyses were done using univariate and multivariate approaches. Both approaches showed that glucose, albumin/globulin, urea, and aspartate aminotransferase concentrations were influenced by the genetic type. Despite at loading Apulo-Calabrese pigs were less reluctant to move and showed a lower vocalization, physiological response to the short distance transport was more intense in this breed when compared with crossbreeds. With regards to meat quality, higher a* and lower L* coordinates were found in Apulo-Calabrese which indicates darker and more reddish meat than crossbreeds. The results from this study may provide insight into the response of local breeds to the transport procedures.

**Abstract:**

Despite the increasing interest in the welfare of animals during transport, very little is known on the response of local pig breeds to the transport procedures. This study aims to compare the effect of short journey on behaviour, blood parameters, and meat quality traits in 51 Apulo-Calabrese and 52 crossbreed [Duroc × (Landrace × Large White)] pigs. All the animals were blood sampled five days before delivery (basal condition) and at exsanguination for the analysis of creatine kinase, cortisol, glucose, lactate, albumin, albumin/globulin, total protein, urea, creatinine, aspartate aminotransferase (AST), alanine aminotransferase, alkaline phosphate, sodium, and potassium. Post mortem pH, color, drip loss, cooking loss, and Warner-Bratzler shear force were measured at different times in *longissimus thoracis* samples. Univariate and multivariate analyses showed that glucose, albumin/globulin, urea, and AST at exsanguination were influenced by the genetic type. Apulo-Calabrese showed the highest increase in blood values of lactate, creatinine, sodium and potassium after the short distance transport. Behavioural occurrences were similar in both genetic types during unloading and lairage. Small differences were observed for meat quality although significantly higher a* and lower L* were found in Apulo-Calabrese pigs, showing meat with a deeper red colour than crossbreeds.

## 1. Introduction

Stress associated with transport has been documented in pigs by a large number of studies [1,2,3]. According to literature, transport stress can cause changes in the behaviour and normal physiological function affecting negatively the welfare of the animals and meat quality attributes [4,5]. It is well known that response to the journey can be influenced by the genotype [6], and factors such as temperature and humidity [7,8], truck conditions [9,10], transport, and/or lairage durations [11] and handling of the animals [12]. Blood parameters at exsanguination have largely been used to assess the stress of transport in livestock [13,14,15].

In pigs, extensive studies on blood parameters after transport have been carried out in conventional commercial breeds and their crossbreeds, whilst little information exists on local pig breeds such as Erhualian [16] and Basque [17].

Among the indigenous Italian pig breeds is Apulo-Calabrese which is included in the list of endangered breeds by the United Nations’ Food and Agriculture Organization [18] and registered in the herd book held by the Italian National Association of Pig Breeders (Associazione Nazionale Allevatori Suini, ANAS). In the year 2017, the breeding population counted 540 sows and 63 boars reared in 63 farms, 31 of which can be found in the Calabria region [19] Apulo-Calabrese is a black-skinned, medium-sized pig with small socks on the forelimbs and large socks on the hind limbs [20]. It is well adaptable to different production systems and can be reared outdoor or indoor in a conventional system [21]. Meat from Apulo-Calabrese is processed into four Protected Designation of Origin (PDO) salami, typical of the Calabrian region [20]. In Italy there is an increasing interest in the welfare of local breeds due to the growing consumer preference for PDO animal friendly products, however, existing research does not provide information on the response of this breed to the transport procedures.

The aim of this study was therefore to investigate the effect of short distance transportation on behavioural response, blood parameters, and meat quality traits of Apulo-Calabrese with respect to crossbreeds.

## 2. Materials and Methods 

### 2.1. Animals

Blood collection at the farm was carried out by a veterinarian in conjunction with routine sampling for sanitary controls. Transportation and slaughter of all pigs were carried out in compliance with EC regulation No 1/2005 and EC regulation No 1099/2013, respectively.

Fifty-one Apulo-Calabrese pigs registered in the herd book of ANAS and 52 crossbreeds [Duroc × (Landrace × Large White)] were used. Apulo-Calabrese pigs were born in this farm from the mating of 13 sows by seven boars of Apulo-Calabrese whilst crossbreeds were bought at about 30 kg live weight from another piggery in the same region. All the pigs were fattened in the same finish facility in separate pens (7–10 pigs per pen) according to their genetic type and were fed the same commercial diet (14,644 KJ DE/kg, 155 g crude protein/kg, 22 g crude fat/kg, 80 g lysine/kg, 58 g ash/kg) in a liquid feeding system with dry feed and water mixed in a 1:4 ratio. All the pigs were identified by a numbered plastic ear tags. Apulo-Calabrese pigs were slaughtered when they reached 135 kg live weight (364 ± 58 days old) due to their slow growth whilst crossbreeds were slaughtered at approximately 155 kg live weight (300–330 days old).

### 2.2. Pre-Slaughter and Slaughter

Approximately 12 h prior to transport, feed was withdrawn. Loading was carried out at 7 a.m. using a mobile ramp (length 4.5 m, width 0.7 m, with solid side walls of 1.0 m and adjustable height) available at the farm. Pigs were delivered through three consignments to the slaughterhouse. At each delivery, pigs from the four pens were herded by electric prods and walked the same distance (25 m) to reach the ramp which was positioned in correspondence with the facility door. The lorry was a hydraulic three tier equipped with internal partition and mechanical ventilation on the left side. Pigs were transported for approximately 1 h to a local processing plant (Piano Lago, Cosenza, Italy) on two decks with a space allowance of about 0.50 m²/100 kg live weight. Unloading was done using the ramp of the lorry and pigs were driven for 10 m to the lairage pens where they rested for 30 min. Outdoor temperature and relative humidity were recorded for each journey by a thermo-hygrometer (mod. HI9065, Hanna, Padua, Italy) during loading and unloading at the entrance of the ramp and at the entrance of the resting pen, respectively (Table 1). During transport and lairage, mixing between pens was avoided. The pigs were stunned by electrical tongs (head only; 220 V, 1.3 A). After stunning, exsanguination blood was collected from each pig (Table 1).

### 2.3. Behavioural Response

The behaviour recordings during loading at the farm and unloading at the abattoir included slipping, falling, reluctance to move, turning back, overlapping, and vocalization as previously described [22,23]. Lying, sitting, and standing behaviours after 25 min of resting time was directly observed for a period of 2 min.

### 2.4. Blood Sampling and Analysis

The animals were blood sampled five days before delivery as reference for basal blood parameter level (T0) and at exsanguination (T1) for the analysis of creatine kinase (CK), cortisol, glucose, lactate, albumin, albumin/globulin ratio, total protein, urea, creatinine, aspartate aminotransferase (AST), alanine aminotransferase (ALT), alkaline phosphate (ALP), sodium (Na^−^), and potassium (K^+^). All biochemical parameters except for glucose, were measured in serum obtained from blood collected in serum separator tubes with gel separator and clot activator (Vacutest Kima, Padua, Italy), let to clot and centrifuged at 1300× *g* at 20 °C (Eppendorf 5702R, Eppendorf, Milan, Italy) for 10 min. Plasma for the measurement of glucose was harvested from anticoagulated blood collected in Na_2_EDTA test tubes containing the glycolysis inhibitor potassium fluoride (Vacutest Kima, Padua, Italy), centrifuged at 1300× *g* at 20 °C for 10 min. Parameters were measured by colorimetric assays on automated analyser (Olympus AU 400, Beckman Coulter, Milan, Italy) with the exception of cortisol. Serum cortisol was determined using the Kit Immulite One Cortisol (medical system code LKC01, Siemens Health Care Diagnostic Limited, Glyn Rhonwy, Gwynedd, UK).

### 2.5. Skin Bruises Measurement

Carcasses were horizontally exsanguinated for 5 min and then hung for 10 min before being submerged in a scalding tank for dehairing at 62 °C for 8 min. After dehairing, skin damages were subjectively assessed by a trained observer, using a four-point scale (1 = none to 4 = severe) based on the scale developed by the Danish Meat Research Institute (DMRI) [24]. The DMRI scale was used to score all skin lesions on the front (head included), middle and hind quarters of each carcass. Moreover, a skin damage score for the whole carcass was calculated using the highest score assigned to each quarter [24]. At about 40 min post mortem, carcasses were split, weighed, and transferred to the chilling room.

### 2.6. Meat Quality Measurements

Measurements of pH on the *longissimus thoracis* muscle (LT) at the level of six/seven thoracic vertebra were recorded at 1, 3, and 6 h post mortem on the left side, with a pH-meter (mod. HI8424, Hanna, Padua, Italy) equipped with Crison electrode (Crison Instruments, SA, Barcelona, Spain) and an automatic temperature compensation probe. At 6 h post mortem the left side was sectioned in the primal cuts and a sample of LT muscle between 6 and 10 thoracic vertebra (10 cm thickness) was collected. After 30 min of blooming, L*, a*, and b* coordinates were determined using Minolta chroma-meter (CR-300, Minolta Camera Co., Ltd., Osaka, Japan) with D65 light source and 0 °C viewing geometry. Samples were transferred (by air) to a laboratory of the Department of Agricultural and Food Sciences (Bologna, Italy). Measurements of pH were repeated at 24 and 72 h post mortem and colour measurements were repeated at 24, 72, and 144 h after slaughter. At the same time interval, cooking loss was determined on a slice of the LT muscle according to Honikel [25] and Warner-Bratzler shear force (WBSF) was measured after cooking using Instron apparatus (mod. 1140, Instron, Norwood, MA, USA). Drip loss was determined at 24 h post mortem [25].

### 2.7. Genotyping

In order to determine the genotype of the Halothane (ryanodine receptor 1, *RYR1*) and Rendement Napole (protein kinase AMP-activated non-catalytic subunit gamma 3, *PRKAG3*), major genes that influence the reactivity of pigs to stress and pork quality, genomic DNA was isolated from blood collected in tubes containing EDTA as anticoagulant. Genotyping of the c.599G > A single nucleotide polymorphism (SNP) *PRKAG3* gene and the g.1843C > T SNP of the *RYR1* gene were done by PCR-RFLP analyses [26,27].

### 2.8. Statistical Analysis

Loading and unloading duration of the two genetic types were compared using T-test. The incidence of pigs showing the different behaviours at loading, unloading and lairage were calculated and the data were processed by Fisher Exact Test procedure of SAS v. 9.3 (SAS Institute, Cary, NC, USA).

Data from blood parameters were transformed to meet assumptions of homogeneity of variance and normality of residuals. Concentrations of CK, lactate, albumin, albumin/globulin ratio, creatinine, and AST were log10 transformed. A square root transformation was used to normalize cortisol and ALT results, while an inverse transformation was used to normalize glucose and K^+^ results. All transformed estimates were back-transformed for presentation to their original scale. Blood parameters were analysed using the mixed model (PROC MIXED of SAS) including the genetic type (two levels), sampling time (two levels: T0 and T1) and their interaction as fixed effects and subject within the day of slaughter as random effect. Sex as a fixed effect and hot carcass weight as a covariate were initially included in the model but they never reached statistical significance (*p* > 0.05) and were removed. Differences between means were tested by the Tukey-Kramer test (*p* < 0.05).

Data of pH, colour, cooking loss and shear force were analysed using PROC MIXED of SAS for repeated measures. The same model for blood parameters was used replacing sampling time factor with measuring time (five levels for pH, four levels for colour, three levels for cooking loss, and shear force). Sex and hot carcass weight did not reach the significant level (*p* > 0.05) and were removed from the model. The data of drip loss was analysed using the same model without measuring time.

In order to highlight possible differences between the two genetic types in blood parameters responses to short distance transport, the variation between blood parameters at exsanguination (T1) and basal blood parameters (T0) has been used to perform an unsupervised multivariate principal component analysis (PCA). All the new variables resulting from the difference between T1 and T0 blood parameters were normally distributed except for cortisol difference, which was root squared transformed in order to meet normal distribution criteria. Furthermore, a PCA has also been used to test the presence of differences in meat quality traits between the two studied genetic types. Unsupervised PCAs have been performed using *ropls* package in the R environment version 3.4.4 [28]. The data were mean centred and unit- variance scaled. The results of multivariate models were plotted on both scores and loadings plot. The combined use of univariate and multivariate analyses was employed in order to test if the results obtained with the multivariate analysis (PCA) were in agreement with what could be observed with the mixed model.

PROC GLIMMIX was used to analyse the effects of genetic type on skin damage scores recorded on each quarter separately as well as on the whole carcass. Because these data approximated a Poisson distribution, the GLIMMIX procedure’s POISSON option was used. The differences in least squares means (L.S.M.) were evaluated using Tukey–Kramer’s test.

## 3. Results and Discussion

Both genetic types did not carry the recessive allele (c.1843T) of the *RYR1* gene [27] and the dominant allele (c.599A or p.200Q) of the *PRKAG3* gene [26] that influence performance and meat quality traits [29,30].

Few research studies have been focused on the effect of transport on local breeds [16,17] although a great deal of literature exists on conventional commercial pigs and their crossbreeds [4,31,32]. The present study reports for the first time the effects of short distance transport on blood parameters and meat quality traits of Apulo-Calabrese. The results obtained showed different physiological response and meat quality attributes in both genetic types after the transport procedure.

### 3.1. Behavioural Recordings and Carcass Bruises

Behavioural occurrences on both genetic types were collected at loading, unloading and lairage. The two genetic types showed no differences in the behavioural occurrences during unloading and lairage, while at loading Apulo-Calabrese pigs showed significantly lower percentages (*p* < 0.05) of reluctance to move and vocalization (Table S1). During lairage, the posture was recorded after 25 min close to the end of the resting time (30 min) as planned routinely by the abattoir. For the duration of time spent in lairage no pigs were observed sitting, 94% of the pigs were lying down and only 6% of the pigs were observed standing. The genetic type did not show significant effect (*p* > 0.05) on skin damage score (whole carcass: 1.14 ± 0.34 and 1.12 ± 0.32 for Apulo-Calabrese and crossbreeds, respectively).

### 3.2. Blood Parameters

Table 2 shows the effects of sampling time, genetic type and their interaction on blood parameters of Apulo-Calabrese and crossbreeds.

Sampling time statistically influenced (*p* < 0.05) all blood parameters, except ALP and sodium whilst the effect of genetic type was significant (*p* < 0.05) for glucose, albumin/globulin ratio, urea, creatinine, AST and potassium. Of particular interest was the interaction between the genetic type and the sampling time since it lays emphasis on the possibility that variation of plasma components between basal and exsanguination is influenced by the genetic type. This interaction was statistically significant (*p* < 0.05) for lactate, albumin/globulin, urea, creatinine, AST, ALT, ALP, sodium, and potassium.

At exsanguination, significantly higher levels of lactate (*p* < 0.05) were found in both genetic types. Higher levels of lactate in the blood have been associated with physical stress [33]. The highest value of lactate in this study was found in Apulo-Calabrese which showed a lower concentration of basal lactate and were driven with less difficulty (minor duration) during loading, as shown in Table 1. According to Broom et al. [34], different breeds cope differently to the handling and transport procedures which could explain the higher levels of lactate found in Apulo-Calabrese in this experiment. Other welfare indicators of stress such as CK and cortisol did not differ between the two genetic types, which is in agreement with the results found by Lebret et al. [17] in the French local Basque and Large White pigs. The similar levels of basal cortisol found in Apulo-Calabrese and crossbreeds in this experiment contrasted with the result by Li et al. [16] who found higher levels of plasma cortisol in Erhualian with respect to Pietrain. Plasma glucose did not differ between both genetic types at exsanguination. However, slightly lower levels of glucose were found in Apulo-Calabrese compared with crossbreeds.

Significantly lower levels (*p* < 0.05) of albumin/globulin were found at exsanguination in Apulo-Calabrese than the crossbreeds, the values obtained were however within the normal physiological range for pigs [35]. With the exception of some globulins, plasma proteins are produced in the liver and are indicators of colloid osmotic pressure of the blood. Lower values of the concentrations may be due to a lack of dietary protein or hepatic damage [35], whilst higher values have been associated with dehydration due to the length of the journey [4].

Higher levels of urea and creatinine in the blood have been associated with food deprivation stress and an increase in physical activity as a result of the transport procedures [36]. In this experiment, significantly lower levels of creatinine (*p* < 0.05) were found in Apulo Calabrese at the basal condition when compared with the crossbreeds whereas serum urea did not differ between the two genetic types. At exsanguination Apulo-Calabrese showed significantly higher levels of urea and lower levels of creatinine when compared with crossbreeds. The values of urea and creatinine obtained in this study were within the normal physiological range for pigs and were in agreement with the results found by Dikic et al. [37] in local Turopolje breed and their crossbreeds [Turopolje × (CHypor × Swedish Landrace)].

AST, ALT, and ALP are chemical indicators of tissue function: elevated levels of these enzymes occur when liver and pancreas are damaged. Significantly higher levels (*p* < 0.05) of AST were found in Apulo-Calabrese at exsanguination when compared with crossbreeds. It is interesting to note that the concentration of AST increased slightly in Apulo-Calabrese from T0 to T1, unlike in the crossbreeds which demonstrated a remarkable increase at slaughter compared with the values obtained at the basal level. According to Pugliese and Sirtori [21] local breeds are reared mostly in an extensive system where pigs forage for food in their surroundings. The elevated levels of AST found in Apulo-Calabrese at the basal condition could be a marker of an overworking hepatic metabolism due to their feeding with formula rations given to conventional fast-growing breeds. Nevertheless, the value obtained was within the range of values found in healthy pigs [38].

Despite the similar levels of ALT found in both genetic types of pigs at exsanguination, there was an increase in this parameter within both genetic types from T0 to T1.

The levels of sodium and potassium found in Apulo-Calabrese at exsanguination were higher when compared with crossbreeds and with values obtained at the basal condition. According to Mota-Rojas et al. [39] transport and slaughter can cause an increase in the concentrations of sodium and potassium, respectively. The values reported in this study were, however, within the normal physiological range for pigs [35].

**Figure 1:**
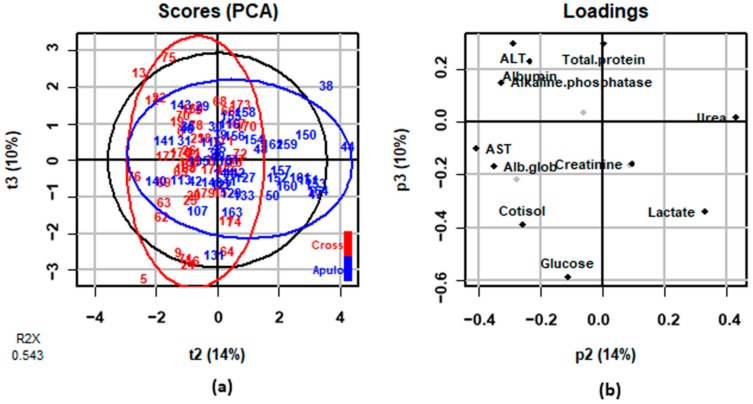
Results of principal components analysis (PCA) on the variations of blood parameters between T1 and T0: (**a**) score plots for principal component 2 (t2) and principal component 3 (t3) of Apulo-Calabrese (blue) and crossbreeds (red) samples; (**b**) loadings plot with the weights of variables included in principal component 2 (p2) and principal component 3 (p3).

The results of the PCA performed on the changes in blood concentrations between T1 and T0 are reported in Figure 1, where the score and the loadings plots are shown. Two samples were not included in this analysis since they appeared to be outliers in orthogonal distance plot. Multivariate analysis generated three Principal Components (PCs: PC1, PC2, and PC3) explained 30%, 14%, and 10% of the total variance, respectively. The two components that explained the most differences between the two genetic types were PC2 and PC3, since plotting these two components samples displayed to be clustered (Figure 1a). The score plot (Figure 1a) presents the graphical projection of the samples into a two-dimensional space with PC2 (t2 in Figure 1a) values as the *x* axis and PC3 (t3 in Figure 1a) as the *y* axis. The red and blue ellipses represent the Mahalanobis distances for the crossbreed and Apulo-Calabrese pigs, respectively, while the black ellipse showed the average area of Mahalanobis distances for the complete population. Figure 1b graphically displays PCA loadings, numerically presented in Table S2. In Figure 1b the variables weighting the most in each PC are displayed: variables which have little contribution to a direction (PC) have almost zero weight (like urea for PC3), while the ones that contribute the most in the definition of the PCs show higher or lower weights (like glucose for PC3). Therefore, the blood parameters that contribute the most in the explanation of the differences among crossbreed and Apulo-Calabrese pigs are grouped together at the opposite quartiles. The results obtained from the PCA were consistent with those observed in the mixed model. Interestingly, the variables that weighted most in PC2 were urea (0.431), AST (−0.406), alb/glob (−0.350), lactate (0.331), and ALP (−0.327) (Table S2), which were the blood parameters that were influenced by the time × genetic type interaction in univariate results. PC3 resulted to be mainly related to glucose (−0.587), which was influenced by the genetic type in the univariate analysis.

### 3.3. Meat Quality Traits

The significance of factors of variations on meat quality parameters of the LT muscle is reported in Table S3. The interaction between measuring time and genetic type was significant for all colour coordinates and for shear force.

**Figure 2:**
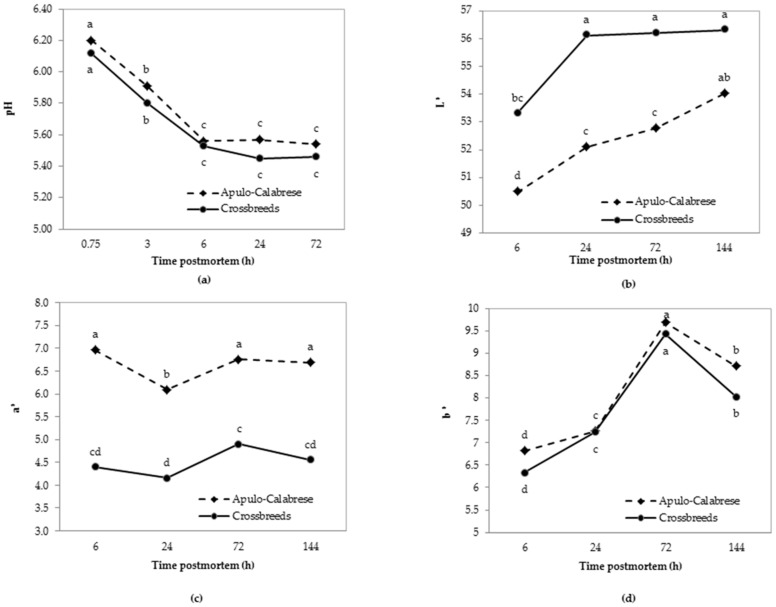
Changes in pH (**a**) and colour coordinates (L* in (**b**), a* in (**c**) and b* in (**d**)) in *longissimus thoracis* in Apulo-Calabrese and crossbreed pigs over time. Different letters in the graphs (a, b, c, d) indicate significant effects (*p* < 0.05).

Post mortem pH decline (Figure 2a) was similar between genetic types although a significant (*p* < 0.05) decrease was reported in the first 6 h after slaughter, which stabilized during subsequent measurements. A similar pH trend was observed by Shen et al. [40] when comparing local Chinese breeds and crossbreeds of pigs.

L* (lightness) measured at 6, 24, 72, and 144 h after slaughter increased progressively as post mortem time increased (Figure 2b). The L* values measured in the meat of Apulo-Calabrese were significantly lower (*p* < 0.05) than those recorded in the meat of crossbreeds in all measuring times, with the exception of those recorded at 144 h after slaughter. The L* coordinate in the meat of crossbreeds stabilized after 24 h whilst that of Apulo-Calabrese maintained an almost constant increase. According to Scheffler and Gerrard [41] post mortem pH can affect muscle colour. Despite the similar levels of pH in this experiment significantly higher (*p* < 0.05) values of a* (Figure 2c) were found in Apulo-Calabrese at each detection time. This indicates that the meat from this breed is distinguished by a deeper red colour like other local European pig breeds [17,42,43]. The trend of the a* coordinate in Apulo-Calabrese pigs showed limited variations and the only value that was significantly different from the others was that measured at 24 h post mortem. The b* value did not differ between the two genetic types as shown in Figure 2d. For both breeds the highest b* values were recorded at 72 h post mortem, while the value decreased significantly at 144 h post mortem.

**Figure 3:**
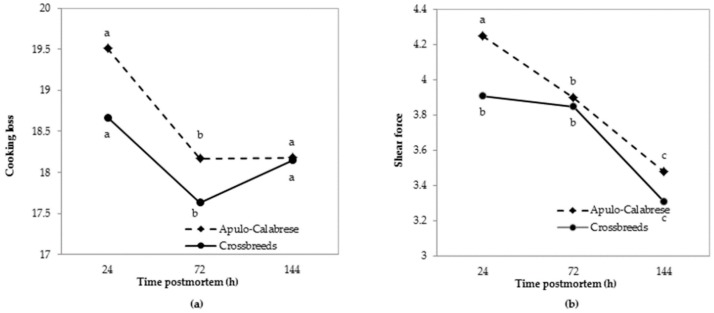
Changes in cooking loss (**a**) and shear force (**b**) in the *longissimus thoracis* in Apulo-Calabrese and crossbreed pigs over time. Different letters in the graphs (a, b, c) indicate significant effects (*p* < 0.05).

Higher values of cooking losses were reported for both genetic types at 24 h post mortem (Figure 3a) compared to lower values recorded at 72 and 144 h after slaughter. Apulo-Calabrese pigs showed slightly higher values at 24 h post mortem compared to crossbreeds, but these differences did not reach statistical significance. Apulo-Calabrese showed lower values of cooking loss when compared with the values of other local breeds, like Cinta Senese [44] and Nero Siciliano [45]. There was no effect of genetic type on drip loss (4.19 ± 0.2 and 4.78 ± 0.2 for Apulo-Calabrese and crossbreeds, respectively).

Warner-Bratzler shear force measured after drip and cooking loss (Figure 3b) decreased as post mortem time increased and showed a similar trend in both genetic types. Nevertheless, higher values were reported for Apulo-Calabrese at 24 and 144 h after slaughter, suggesting the need to subject the meat of Apulo-Calabrese to ageing if it is intended for fresh consumption.

**Figure 4:**
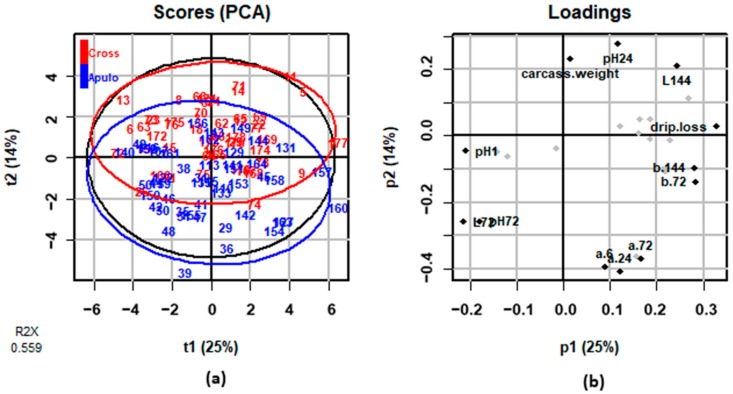
Results of principal components analysis (PCA) on the meat quality traits: (**a**) score plots for principal component 1 (t1) and principal component 2 (t2) of Apulo-Calabrese (blue) and crossbreeds (red) samples; (**b**) loadings plot with the weights of variables included in principal component 1 (p1) and principal component 2 (p2).

Figure 4 reports the results of the PCA performed on meat quality traits. Multivariate analysis generated four PCs: PC1, PC2, PC3, and PC4 explained 25%, 14%, 9%, and 8% of the total variance, respectively. The component that explained the most differences between the two genetic types was PC2, since samples displayed to be clustered for PC2 (Figure 4a). Figure 4b graphically displays PCA loadings, numerically presented in Table S4.

The variables that weighted most in PC2 were colour coordinates a* at 24 h (−0.409), a* at 6 h (−0.394), a* at 72 h (−0.368), a* at 144 h (−0.363), L* at 72 h (−0.257), and pH measured at 24 h (0.276). The high weights observed for a* colour coordinate are in agreement with the significant differences obtained from univariate analysis reported in Figure 2c, where redness-greenness value a* was highly divergent at all the measuring times between the two pig genetic types. Together with a*, other variables that contribute in differentiating the two genetic types were L* and pH, as can be noticed both by PCA loadings in Figure 4b and Table S4 and by mixed model results in Table S3. Interestingly, despite the different statistical assumptions of mixed and multivariate analysis, the results obtained are quite concordant, highlighting that colour coordinates represents the meat quality attributes discriminating the most the two genetic types. Anyway, PCA results suggested that, when considering together all the meat quality variables and taking into account their correlated nature, also pH measured at 24 h has a consistent weight in differentiating Apulo-Calabrese from crossbreed pigs. This result may also be noticed in Table S3, from the mixed model results. Despite the genetic type had not a significant effect on *longissimus thoracis* pH, the estimated L.S.M. for pH at 24 h were the most divergent between the two genetic types (5.57 for Apulo-Calabrese and 5.45 for crossbreeds) when compared with the pH measured at the other times. This result suggests that using a combined statistical approach may allow to highlight the main differences that would have not been appreciable with the use of univariate statistics alone.

## 4. Conclusions

Overall, the results obtained in this study broaden the knowledge on the Apulo-Calabrese pig breed, which showed higher levels of lactate, urea and AST after short distance transport indicating a more intense physiological response when compared with crossbreeds. With regards to meat quality, similar trends for pH, drip loss and cooking loss were found for both genetic types. The higher a* coordinate found in Apulo-Calabrese pig indicates that meat from this breed has a deeper red colour and can be used for the production of typical cured meat which on the basis of the gathered evidence could be produced without the use of additives intended to improve colour. The results in this preliminary study may provide insight into the response of local breeds to the transport process. However, due to practical restraints, in this pilot research it was not possible to investigate the effects of different pre-slaughter treatments on the behavioural and physiological responses of the two genetic types, further research is needed to evaluate the effect of different transport durations and handling practices on welfare and meat quality traits when transporting local pig breeds.

## Figures and Tables

**Table 1:** Outdoor temperature (Temp), relative humidity (RH), and durations of loading, transport and unloading recorded in pigs in the three deliveries.

Delivery	Genetic Type	Number of Pigs	Loading			Transport	Unloading		
			Duration ^1^ (min)	Temp (°C)	RH (%)	Duration (min)	Duration ^2^ (min)	Temp (°C)	RH (%)
1	Apulo-Calabrese	19	8	10.3	79	63	2	11.2	88.4
	Crossbreed	20	16				9		
2	Apulo-Calabrese	17	10	18	72.3	67	7	19.5	65.8
	Crossbreed	17	16				3		
3	Apulo-Calabrese	15	6	21.5	59.6	60	5	19.7	65.5
	Crossbreed	15	6				2		

^1^ From the opening of the farm gate until the last pig entered the lorry. ^2^ From the opening of the gate of the lorry until the last pig entered the lairage pen.

**Table 2:** Effects of sampling time (T), genetic type (GT), and their interaction (T × GT) on least square means (L.S.M) and standard error of means (S.E.M.) of blood parameters of Apulo-Calabrese and crossbreed pigs on farm (baseline) and at exsanguination.

Blood Parameters	Baseline		Exsanguination					
	Apulo-Calabrese	Crossbreed	Apulo-Calabrese	Crossbreed		*p*-Values		
	L.S.M				S.E.M	T	GT	T × GT
Creatine Kinase, CK (U/L)	954.99	831.76	2089.30	2187.76	0.05	<0.0001	0.7529	0.2130
Cortisol (mg/dL)	16.72	17.38	56.31	56.73	0.26	<0.0001	0.8730	0.8690
Glucose (mg/dL)	71.23	78.13	100.57	114.59	0.00	<0.0001	0.0004	0.9497
Lactate (mg/dL)	24.23 ^c^	33.85 ^b^	181.01 ^a^	143.81 ^a^	0.03	<0.0001	0.4549	<0.0001
Albumin (g/dL)	3.47	3.55	3.63	3.80	0.00	<0.0001	0.1747	0.2862
Albumin/globulin, Alb/glob	0.93 ^a^	0.99 ^a^	0.82 ^b^	0.95 ^a^	0.01	<0.0001	0.0031	<0.0001
Total protein (g/dL)	7.30	7.20	8.15	7.84	0.10	<0.0001	0.0686	0.0674
Urea (mg/dL)	37.71 ^a^	36.91 ^a^	37.55 ^a^	33.50 ^b^	0.96	0.0103	0.0404	0.0185
Creatinine (mg/dL)	1.65 ^c^	1.89 ^b^	2.08 ^a^	2.25 ^a^	0.00	<0.0001	<0.0001	<0.0001
Aspartate aminotransferase, AST (U/L)	70.80 ^a^	33.25 ^c^	77.73 ^a^	54.29 ^b^	0.03	<0.0001	<0.0001	<0.0001
Alanine aminotranferase, ALT (U/L)	54.83 ^a^	52.51 ^b^	58.52 ^ab^	59.41 ^ab^	0.11	<0.0001	0.7309	0.0357
Alkaline phosphatase, ALP (U/L)	145.14 ^a^	118.12 ^b^	134.57 ^ab^	119.03 ^b^	5.69	0.1105	0.0527	0.0124
Sodium, Na (mEq/L)	139.03 ^b^	142.00 ^b^	147.97 ^a^	145.77 ^a^	1.18	0.7937	0.7937	0.0017
Potassium, K (mEq/L)	5.34 ^c^	5.74 ^b^	6.93 ^a^	6.20 ^ab^	0.00	<0.0001	0.0017	0.0004

Means on the same row with different superscript letters (^a^, ^b^, **^c^**) indicate significant effects (*p* < 0.05) of the interaction between sampling time (T) and genetic type (GT).

## Supplementary Materials

The following are available online at at http://www.mdpi.com/2076-2615/8/10/177/s1. Table S1: Percentages of Apulo-Calabrese and Crossbreed pigs showing the different behaviours during loading, unloading, and lairage with the P values of the differences between percentages; Table S2: Loadings of the PCA performed on blood parameters; Table S3: Significance of factors of variations included in the model used for meat quality traits; Table S4: Loadings of the PCA performed on meat quality traits.
