# Supplementary material for: Apulo-Calabrese and Crossbreed Pigs Show Different Physiological Response and Meat Quality Traits after Short Distance Transport
Source: Animals (Basel). 2018 Oct 10;8(10):177. doi: 10.3390/ani8100177 (PMC6210385; doi:10.3390/ani8100177)
Supplement: Supplementary file 1 [file animals-08-00177-s001.docx]

**Supplementary Materials**

**Table S1**. Percentages of Apulo-Calabrese and crossbreed pigs showing the different behaviours during loading, unloading, and lairage with the P values of the differences between percentages.

| **Check point** | **Measurement** |  | **Apulo-Calabrese (%)** | **Crossbreed (%)** | **P**  **(Fisher Exact Test)** |
| --- | --- | --- | --- | --- | --- |
| Loading | Slipping |  | 2 | 8 | 0.363 |
|  | Falling |  | 2 | 2 | 0.980 |
|  | Reluctance to move |  | 10 | 34 | 0.004 |
|  | Turning back |  | 6 | 6 | 0.980 |
|  | Overlapping |  | 10 | 16 | 0.555 |
|  | Vocalization |  | 12 | 38 | 0.003 |
|  |  |  |  |  |  |
| Unloading | Slipping |  | 2 | 6 | 0.618 |
|  | Falling |  | 0 | 0 | --- |
|  | Reluctance to move |  | 2 | 10 | 0.205 |
|  | Turning back |  | 2 | 8 | 0.618 |
|  | Overlapping |  | 0 | 6 | 0.243 |
|  | Vocalization |  | 0 | 10 | 0.057 |
|  |  |  |  |  |  |
| Lairage | Lying |  | 94 | 94 | 0.984 |
|  | Sitting |  | 0 | 0 | -- |
|  | Standing |  | 6 | 6 | 0.984 |

**Table S2.** Loadings of the PCA performed on blood parameters.

|  | **PCA loadings** | | |
| --- | --- | --- | --- |
| **Blood parameters** | **PC1** | **PC2** | **PC3** |
| Creatine Kinase, CK (U/L) | 0.005 | –0.274 | –0.218 |
| Cortisol (mg/dL) | 0.125 | –0.256 | –0.389 |
| Glucose (mg/dL) | 0.134 | –0.109 | –0.587 |
| Lactate (mg/dL) | 0.261 | 0.331 | –0.340 |
| Albumin (g/dL) | 0.397 | –0.236 | 0.229 |
| Albumin/globulin, Alb/glob | –0.055 | –0.350 | –0.168 |
| Total protein (g/dL) | 0.430 | 0.006 | 0.295 |
| Urea (mg/dL) | 0.145 | 0.431 | 0.017 |
| Creatinine (mg/dL) | 0.389 | 0.094 | –0.157 |
| Aspartate aminotransferase, AST (U/L) | –0.050 | –0.406 | –0.099 |
| Alanine aminotransferase, ALT (U/L) | 0.272 | –0.286 | 0.296 |
| Alkaline phosphatase, ALP (U/L) | 0.048 | –0.327 | 0.151 |
| Sodium, Na (mEq/L) | 0.412 | –0.061 | 0.036 |
| Potassium, K (mEq/L) | 0.363 | 0.089 | –0.162 |

**Table S3.** Effects of measuring time (T), genetic type (GT) and their interaction (T x GT) on least square means (L.S.M.) and standard error of means (S.E.M) of meat quality traits of Apulo-Calabrese and crossbreed pigs

| **Meat quality traits** | **Apulo-Calabrese** | | | | | | **Crossbreed** | | | | | | |  | **T** | | **GT** | **T x GT** | | |  |
| --- | --- | --- | --- | --- | --- | --- | --- | --- | --- | --- | --- | --- | --- | --- | --- | --- | --- | --- | --- | --- | --- |
|  | **L.S.M. at different time *postmortem* (h)** | | | | | | | | | | | | | **S.E.M.** |  |  |  |  |  |  |  |
|  | **0.75** | **3** | **6** | **24** | **72** | **144** |  | **0.75** | **3** | **6** | **24** | **72** | **144** |  |  |  |  |  |  |  |  |
| pH | 6.20 | 5.91 | 5.56 | 5.57 | 5.54 | - |  | 6.12 | 5.80 | 5.53 | 5.45 | 5.46 | - | 0.0256 | <0.0001 | 0.0583 | | | 0.5221 |  |  |
| L* (brightness) | - | - | 50.5 | 52.1 | 52.8 | 54.0 |  | - | - | 53.3 | 56.1 | 56.2 | 56.3 | 0.6064 | <0.0001 | <.0001 | | | 0.0095 | | |
| a* (redness) | - | - | 7.00 | 6.10 | 6.80 | 6.70 |  | - | - | 4.4 | 4.20 | 4.90 | 4.60 | 0.2663 | <0.0001 | <.0001 | | | 0.0359 | | |
| b* (yellowness) | - | - | 6.82 | 7.26 | 9.69 | 8.72 |  | - | - | 6.33 | 7.24 | 9.43 | 8.03 | 0.2269 | <0.0001 | 0.2403 | | | 0.0316 | | |
| Drip loss (%) | - | - | - | 4.16 | - | - |  | - | - | - | 4.78 | - | - | 0.2296 | - | 0.3568 | | | - | | |
| Cooking loss (%) | - | - | - | 19.51 | 18.17 | 18.18 |  | - | - | - | 18.67 | 17.63 | 18.15 | 0.5186 | 0.0286 | 0.2089 | | | 0.1896 | | |
| Shear force (N) | - | - | - | 4.25 | 3.90 | 3.48 |  | - | - | - | 3.91 | 3.85 | 3.31 | 0.0702 | <.0001 | 0.0645 | | | <.0001 | | |

- : not measured

**Table S4:** Loadings of the PCA performed on meat quality traits.

|  | **PCA Loadings** | | | |
| --- | --- | --- | --- | --- |
|  | **PC1** | **PC2** | **PC3** | **PC4** |
| Carcass weight | 0.016 | 0.231 | 0.110 | 0.295 |
| pH 1 h | –0.209 | –0.045 | 0.057 | 0.196 |
| pH 3 h | 0.186 | 0.050 | –0.154 | –0.292 |
| pH 6 h | 0.228 | –0.012 | –0.102 | –0.364 |
| pH 24 h | 0.118 | 0.276 | 0.278 | –0.140 |
| pH 72 h | –0.179 | –0.256 | –0.227 | 0.061 |
| L* 6 h | 0.270 | 0.113 | –0.159 | –0.133 |
| L* 24 h | 0.269 | 0.164 | 0.174 | –0.157 |
| L* 72 h | –0.214 | –0.257 | 0.054 | –0.114 |
| L* 144 h | 0.244 | 0.210 | –0.181 | 0.091 |
| a* 6 h | 0.090 | –0.394 | 0.007 | –0.074 |
| a*24 h | 0.122 | –0.409 | 0.104 | –0.043 |
| a* 72 h | 0.165 | –0.368 | 0.103 | 0.159 |
| a* 144 h | 0.156 | –0.363 | 0.077 | 0.111 |
| b* 6 h | 0.219 | –0.107 | –0.257 | –0.148 |
| b* 24 h | 0.214 | –0.098 | 0.393 | –0.171 |
| b* 72 h | 0.284 | –0.137 | 0.080 | 0.183 |
| b* 144 h | 0.281 | –0.095 | –0.023 | 0.084 |
| Cooking loss 24 h | 0.185 | –0.011 | –0.157 | 0.344 |
| Cooking loss 72 h | 0.162 | 0.007 | –0.323 | 0.085 |
| Cooking loss 144 h | 0.122 | 0.030 | 0.137 | 0.031 |
| Shear force 24 h | –0.014 | –0.037 | –0.110 | –0.340 |
| Shear force 72 h | –0.142 | –0.049 | –0.312 | –0.143 |
| Shear force 144 h | –0.119 | –0.063 | –0.267 | –0.263 |
| Drip loss | 0.329 | 0.029 | 0.015 | 0.011 |
